# Safety and Immunogenicity of a Novel Recombinant Simian Adenovirus ChAdOx2 as a Vectored Vaccine

**DOI:** 10.3390/vaccines7020040

**Published:** 2019-05-15

**Authors:** Pedro M. Folegatti, Duncan Bellamy, Rachel Roberts, Jonathan Powlson, Nick J. Edwards, Catherine F. Mair, Georgina Bowyer, Ian Poulton, Celia H. Mitton, Nicky Green, Eleanor Berrie, Alison M. Lawrie, Adrian V.S. Hill, Katie J. Ewer, John Hermon-Taylor, Sarah C. Gilbert

**Affiliations:** 1 The Jenner Institute, University of Oxford, ORCRB, Roosevelt Drive, Oxford OX3 7DQ, UK; duncan.bellamy@ndm.ox.ac.uk (D.B.); rachel.roberts@ndm.ox.ac.uk (R.R.); jpowlson@gmail.com (J.P.); nick.edwards@ndm.ox.ac.uk (N.J.E.); 2177110M@student.gla.ac.uk (C.F.M.); georgina.bowyer@ndm.ox.ac.uk (G.B.); ian.poulton@ndm.ox.ac.uk (I.P.); celia.mitton@ndm.ox.ac.uk (C.H.M.); alison.lawrie@ndm.ox.ac.uk (A.M.L.); adrian.hill@ndm.ox.ac.uk (A.V.S.H.); katie.ewer@ndm.ox.ac.uk (K.J.E.); sarah.gilbert@ndm.ox.ac.uk (S.C.G.); 2 Clinical BioManufacturing Facility, Churchill Hospital, University of Oxford, Oxford OX3 7JT, UK; nicky.green@ndm.ox.ac.uk (N.G.); eleanor.berrie@ndm.ox.ac.uk (E.B.); 3 Department of Nutritional Sciences, Faculty of Life Sciences & Medicine, Franklin Wilkins Building, King’s College London, London SE1 9NH, UK; j.hermon@kcl.ac.uk

**Keywords:** viral vector, vaccine, T-cell, *Mycobacterium avium* subsp. *paratuberculosis*

## Abstract

Adenovirus vectored vaccines are a highly effective strategy to induce cellular immune responses which are particularly effective against intracellular pathogens. Recombinant simian adenovirus vectors were developed to circumvent the limitations imposed by the use of human adenoviruses due to widespread seroprevalence of neutralising antibodies. We have constructed a replication deficient simian adenovirus-vectored vaccine (ChAdOx2) expressing 4 genes from the *Mycobacterium avium* subspecies *paratuberculosis* (*AhpC*, *Gsd*, *p12* and *mpa*). Safety and T-cell immunogenicity results of the first clinical use of the ChAdOx2 vector are presented here. The trial was conducted using a ‘three-plus-three’ dose escalation study design. We demonstrate the vaccine is safe, well tolerated and immunogenic.

## 1. Introduction

Viral vectored vaccines are known to be an effective mechanism to induce cellular responses compared with subunit vaccines and can induce protective T cell responses against intracellular pathogens [1]. Recombinant viruses are excellent vehicles for vaccine delivery as viral proteins can act as potent adjuvants and can directly infect antigen-presenting cells [2]. Adenoviruses are highly attractive vectors for human vaccination as they possess a stable genome which prevents inserts of foreign genes from being deleted and they can infect large numbers of cells without any evidence of insertional mutagenesis [3].

Human adenovirus vectors have been used extensively in clinical trials with an excellent safety profile, mainly as vectors for Human Immunodeficiency Virus (HIV) vaccines [4,5,6,7,8,9,10,11,12,13,14,15,16,17]. However, the ubiquity of human adenovirus infections generates levels of host anti-vector immunity that may limit the utility of this vector [18]. Pre-existing immunity can be circumvented by employing vectors based on closely related simian adenoviruses, which have significantly lower seroprevalence rates in human populations [19,20]. Replication-deficient adenovirus vectors may be produced by removing the E1 gene from the viral genome, which is essential for viral replication, and supplying the E1 gene product via the production cell line. The E3 gene may also be deleted, which increases the space available within the viral genome for the addition of the required antigen target downstream of a mammalian promoter to direct expression of the vaccine insert inside a transduced cell after vaccination [21].

*Mycobacterium avium* subspecies *paratuberculosis* (MAP) is a slow growing, fastidious, gram-positive, acid-fast, non-chromogen microorganism of 0.5–1.5 µm [22]. It is known to be the causative agent of chronic granulomatous enteritis in ruminants (Johne’s Disease) and its zoonotic potential has been suggested since 1913, when the similarities between two chronic inflammatory bowel diseases was first described: Johne’s Disease in cattle and what would later be defined as Crohn’s Disease (CD) in humans [23,24,25]. MAP is widespread in the environment as a result of shedding in the faeces of infected ruminants. The presence of farm animal reservoirs and large scale environmental contamination leads to inevitable human population exposures, not only through the food chain, but also through contaminated soil, water supplies and aerosols [26,27].

Crohn’s Disease is a debilitating chronic inflammatory bowel condition of unknown aetiology and multifactorial pathogenesis, with complex interactions between genetic and environmental factors [28,29]. MAP infection has been suggested as a potential causative agent of CD and there is now an established association between MAP infection and Crohn’s disease, although questions on its exact role remain unanswered [30,31]. MAP has also been associated with, and is hypothesized to play a role in, other auto-immune diseases, such as type-1 diabetes and multiple sclerosis [32,33].

The first clinical trial of the recombinant chimpanzee adenovirus vector ChAdOx2 expressing genes from MAP is described here. The vaccine antigen consists of a fusion construct from the *AhpC*, *Gsd*, *p12* and *mpa* MAP genes, which are present in all MAP strains. The antigen was named HAV and has been described elsewhere [34]. Safety and cellular immunogenicity were assessed in healthy adult volunteers receiving a single dose of the vaccine.

## 2. Materials and Methods

### 2.1. Study Design And Participants

This first-in-human, non-randomised, open-labelled, dose escalation, phase I trial was reviewed and approved within the UK by the Medicines and Healthcare Products Regulatory Agency and the Regional Ethics Committee and it is registered at Clinicaltrials.gov (identifier: NCT03027193).

**Figure 1:**
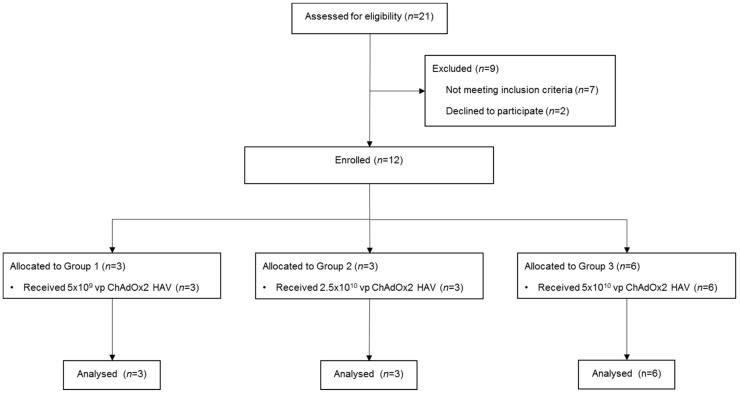
CONSORT diagram. Vp, viral particles.

Male and female healthy adult volunteers aged 18–50 years were recruited and enrolled into 3 groups (Figure 1, CONSORT diagram) at the Centre for Clinical Vaccinology and Tropical Medicine, Churchill Hospital, Oxford, UK. Written informed consent was obtained in all cases and the trial was performed according to the principals of the Declaration of Helsinki.

Volunteers were enrolled and doses escalated according to a ‘three-plus-three’ study design, as previously described [35]. All vaccinations were given intramuscularly into the deltoid. The first volunteer was vaccinated with 5 × 10^9^ viral particles (vp) of ChAdOx2 HAV. No other volunteers were vaccinated until 48 hours had elapsed following this first vaccination. No severe or serious adverse reactions occurred and, therefore, a further two volunteers were vaccinated with the 5 × 10^9^ vp dose. Following review of the safety data at day 7 post vaccination, the profile of adverse reactions was found to be acceptable and the first participant in the next incremental dose group was enrolled (2.5 × 10^10^ vp ChAdOx2 HAV). Doses were gradually increased up to 5 × 10^10^ vp ChAdOx2 HAV following the same procedures, aiming to provide an optimal dose considering the tolerability, reactogenicity and immunogenicity profiles.

Three participants received ChAdOx2 HAV at 5 × 10^9^ vp and another 3 volunteers were vaccinated at 2.5 × 10^10^ vp. As none of the participants presented either severe or serious adverse reactions, further participants were vaccinated at the 5 × 10^10^ vp dose. Given the favourable safety profile, the top dose group was expanded to a maximum of 6 volunteers to generate additional safety and immunogenicity data.

Six participants had previously received an adenoviral vectored vaccine more than 12 months before enrolment, as part of clinical trials for Malaria and Ebola candidate vaccines. Five of those were previously given a chimpanzee adenovirus serotype 63 vectored vaccine (ChAd63) and one participant had a chimpanzee adenovirus serotype 3 vectored vaccine (ChAd3).

Blood samples were taken at pre-defined time points for to assess haematological and biochemical parameters and to assess immunogenicity. In all cases, the vaccine was administered in the deltoid muscle and observations were taken up to 1 hour after vaccination. Volunteers were followed for 2 months and completed electronic diaries regarding adverse events for 4 weeks following vaccination.

The primary objective was to assess safety and reactogenicity measured as: (a) occurrence of solicited local reactogenicity signs and symptoms for 7 days following the vaccination; (b) occurrence of solicited systemic reactogenicity signs and symptoms for 7 days following the vaccination; (c) occurrence of unsolicited adverse events for 28 days following vaccination; (d) change from baseline for safety laboratory measures and; (e) occurrence of serious adverse events during the whole study duration.

The secondary objective was to assess cellular immunogenicity of ChAdOx2 HAV measured by interferon-γ (IFNγ) enzyme-linked immunospot (ELISpot).

### 2.2. Design and Construction of ChAdOx2 HAV Vaccine

The ChAdOx2 HAV vaccine consists of the replication-deficient simian adenovirus vector ChAdOx2 [36], containing the 95kDa fusion construct from 4 MAP genes present in all MAP strains, named HAV: 1589c (*AhpC*), MAP 1234 (*Gsd*), 2444c (*p12*) and 1235 (*mpa*) [34].

The replication-defective E1/E3 deleted chimpanzee adenovirus vector ChAdOx2 was produced starting from the wild-type replication-competent isolate AdC68 (species adenovirus E, also known as SAdV-25 and Pan 9), with further modification to the E4 region. The vector was constructed in a bacterial artificial chromosome (BAC) to facilitate genetic manipulation of genomic clones with improved stability and flexibility. To generate a molecular clone of the AdC68 genome, a BAC gap repair vector was constructed containing Polymerase Chain Reaction (PCR)-amplified regions of homology to the left and right flanks of the viral genome as described elsewhere [37]. An extra homology flank downstream of the adenovirus E1 region was included to enable deletion of E1 and placement of a unique restriction site at the E1 locus, concomitant with genomic insertion into the BAC. The E1 region is essential for viral replication, hence the ability to delete E1 at this stage renders the new vector immediately replication incompetent. Replication incompetent (E1-deleted) clones were successfully identified by PCR screening and transfection into E1 complementing human embryonic kidney 293 (HEK293) cells confirmed the ability of all candidate clones of the new vector to generate infectious virions. Galactokinase (GalK) recombineering was used to delete the non-essential adenovirus E3 region, which increases the insert capacity of the new vector by approximately 5kb. Proteins encoded by the E4 region of adenoviruses interact with E1 during viral replication. The imperfect interaction between the gene products of the AdHu5 E1 gene produced by HEK293 cells and simian E4 gene products has been found to result in impaired viral replication in this cell line and consequently lower virus yields. In the construction of ChAdOx2, the whole of the native AdC68 E4 region was replaced with the E4Orf1, E4Orf2 and E4Orf3 coding regions from AdY25 and the E4Orf4, E4Orf6 and E4Orf6/7 coding regions from AdHu5.

Expression of the HAV insert is driven by the human cytomegalovirus immediate early promoter, and insertion of recombinant antigens at the E1 locus was performed using Gateway site specific recombination technology (Invitrogen/Thermo Fisher, Waltham, Massachusetts, USA).

ChAdOx2 HAV was manufactured to clinical good manufacturing practice (cGMP) by the Clinical Biomanufacturing Facility (University of Oxford, Oxford, UK) in the HEK293 cell line. The vectored vaccine was purified and sterile filtered to generate a clinical lot at a concentration of 1.6 × 10^11^ vp per mL.

### 2.3. IFN-γ ELISpot

Responses to vaccination with ChAdOx2 HAV were assessed by IFN-γ ELISpot assays using freshly-isolated peripheral blood mononuclear cells (PBMC) stimulated with pools of peptides spanning the HAV vaccine construct. Assays were performed prior to vaccination (day 0) and at one and two months post vaccination (days 28 and 56). Methodology was as described previously [38] except that 2.5 × 10^5^ PBMCs were assayed per well. Results are expressed as spot forming units (SFU) per million PBMCs, calculated by subtracting the mean negative control response from the mean of each peptide pool response and then summing the response for the eight peptide pools. Each pool contained between 11 and 13–15 mer peptides overlapping by 10 amino acids, spanning the complete vaccine insert. Peptides were pooled so that no pool contained peptides from more than one antigen in the insert. ELISpot plates were excluded if responses were >80 SFU/million PBMC in the negative control (medium only wells) or <800 SFU/million PBMC in the positive control (phytohemagglutinin/staphylococcal enterotoxin B) wells. These quality control (QC) criteria were defined prior to the commencement of sample analysis and only one sample (a D56 assay in the middle dose group) was removed from the dataset due to high background in the negative control wells.

### 2.4. Statistical Analysis

Safety end points are described as frequencies and percentages alongside their 95% confidence intervals. Statistical analysis of immunogenicity data was conducted using GraphPad Prism version 7.03 for Windows (GraphPad Software Inc., California, USA). Median and interquartile range are given for each parameter. Comparisons between time points and baseline were performed using non-parametric tests, with an alpha value <0.05 considered significant.

### 2.5. Anti-Vector Neutralising Antibodies (NAb)

NAb titers to the ChAdOx2 and ChAdOx1 vectors were measured using a secreted embryonic alkaline phosphatase-reporter (SEAP) assay, as previously described [39] which measures the reciprocal of the serum dilution required to reduce in vitro expression of vector-expressed SEAP by 50%, 24 hours post transduction. ChAdOx1 is a related adenovirus vector produced from the wildtype Y25 vector, which has been previously employed as a vector for influenza A, tuberculosis, and Rift Valley fever virus vaccine candidates [35,39,40,41]. SEAP-expressing ChAdOx2 or ChAdOx1 was pre-incubated with an equal volume of serially diluted, heat inactivated (56 °C for 90 minutes) human sera (dilutions of 1:18, 1:72, 1:288, 1:1152 and 1:4608) for 1 hour at 37 °C. Pooled sera with a known high neutralizing titre (greater than 1000) and individual sera with known neutralising titres (200–500), plus known unreactive sera, were used as positive and negative controls respectively. Following incubation, 200 μL serum: Virus dilutions were used to transduce GripTite 293 Macrophage Scavenger receptor (MSR) cells (Invitrogen, Carlsbad, CA), seeded in 96-well plates at 3 × 10^4^ cells/well. Cell supernatants were collected 24 hours post transduction, and SEAP concentration was quantified using the Tropix PhosphaLite Chemiluminescent Assay Kit (Applied Biosystems, Warrington, UK). Luminescence was measured using a Varioskan flash luminometer (Thermo Scientific, Loughborough, UK). Neutralizing titers were expressed as the reciprocal of the serum dilution required to reduce SEAP expression by 50%, 24 hours post transduction, by linear interpolation from adjacent values.

## 3. Results

### 3.1. Study Population

Twelve male and female healthy adult volunteers aged 18–50 years were enrolled into 3 groups and received a single dose of ChAdOx2 HAV (CONSORT diagram), using a dose escalation approach as described in the Methods section. The median age of participants was 39 years (range 21–49) and 66.67% were females.

Volunteers were vaccinated at the Centre for Clinical Vaccinology and Tropical Medicine, Oxford, UK from March until June 2017. All participants attended a series of follow-up visits for safety and immunology purposes up to 2 months post vaccination. Long term follow-up for serious adverse events was conducted for 12 months after enrolment.

### 3.2. Vaccine Safety

ChAdOx2 HAV was well tolerated at all dose groups. A total of 40 adverse events (AEs) considered possibly, probably or definitively related with the study vaccine were reported. Of those 15 (37.50%) were local and 25 (65.50%) were systemic AEs.

**Figure 2:**
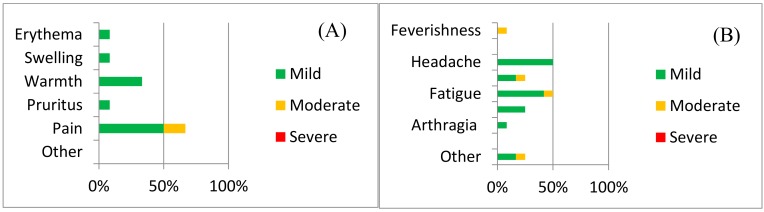
Safety data for ChAdOx2 HAV: The frequency of adverse reactions following vaccination with ChAdOx2 HAV is shown, with severity indicated by shading. (**A**) Local adverse reactions and (**B**) systemic adverse reactions. Data represent adverse reactions from all 12 volunteers across all three doses.

Local adverse reactions included pain, redness, itching, and warmth (Figure 2a). Injection site pain was the most common local AE experienced by 66.67% (39.06–86.19 95%CI) of the 12 volunteers vaccinated and were more common amongst volunteers receiving the higher dose (group 3) than amongst the participants in groups 1 and 2 (OR 10.0, 0.65–154.4 95%CI).

Headache and fatigue (50%, 25.38–74.62% 95%CI) were the most common systemic adverse reactions. Other systemic AEs reported included subjective feverishness, malaise, myalgia and arthralgia (Figure 2b and Supplementary Table S1). A breakdown of adverse reactions by dose is provided in the Supplementary Material (Supplementary Figures S1–S6). Laboratory AEs are presented in Supplementary Table S2.

There were no serious adverse reactions following vaccination with ChAdOx2 HAV, at any given dose. Neither objective fever nor severe AEs considered to be at least possibly related with the vaccine were reported.

The vast majority of AEs were mild in nature (85%, 70.93–92.94% 95%CI) and all were self-limited. Pain at injection site resolved within 4 days and all other AEs resolved within 24–72 h.

### 3.3. Immunogenicity

**Figure 3:**
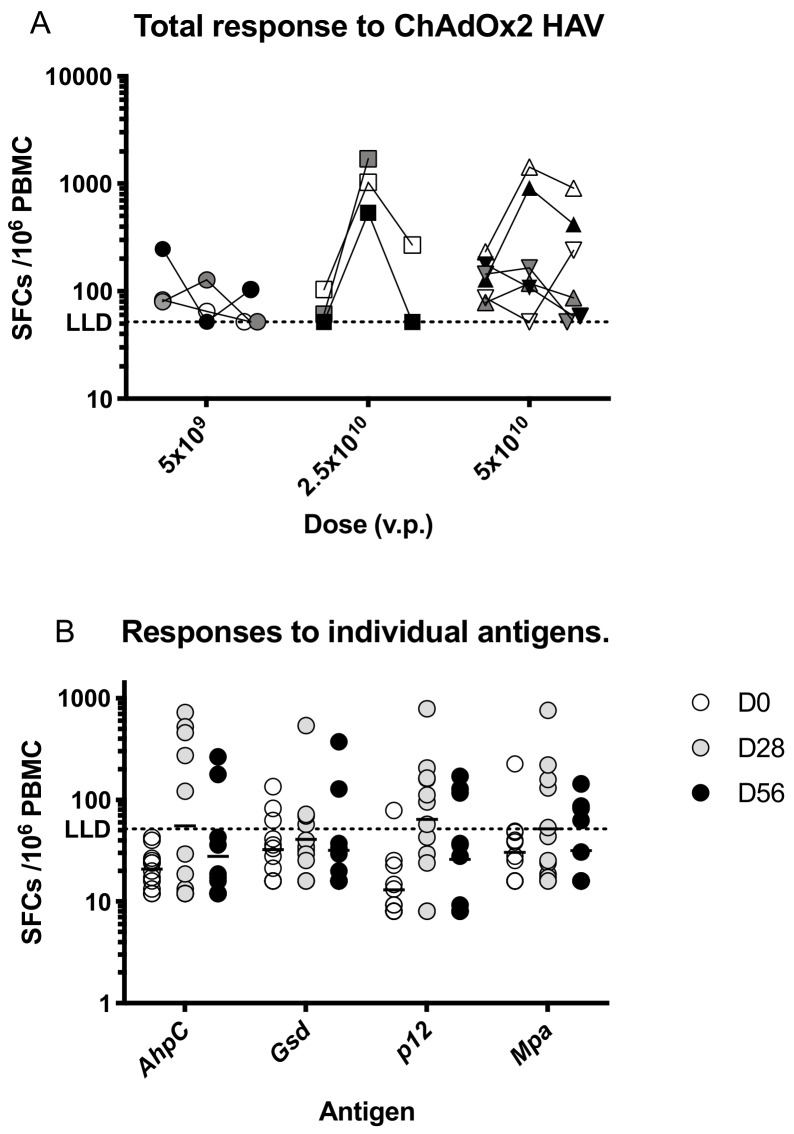
T cell responses to ChAdOx2 HAV. (**A**) Summed interferon-γ (IFNγ) enzyme-linked immunospot (ELISpot) responses to the vaccine insert stratified by dose and time point. Prior to vaccination, responses to HAV antigens were low, with a geometric mean response of 109 (95% CI 79–151) spot-forming cells (SFC) per million PBMC. Responses increased significantly at day 28 in the group that received 2.5 × 10^10^ vp compared with responses in all participants at day 0 (*p* < 0.05, Kruskal-Wallis test with Dunn’s correction for multiple comparisons). No other comparisons were statistically significant. (**B**) Responses to individual antigens encoded in the vaccine insert at each time point. Lines represent geometric means. Vp, viral particles. LLD, lower limit of detection for the ELISPOT assay.

Prior to vaccination, responses to HAV antigens were low, with a geometric mean response of 109 (95% CI 79–151) spot-forming cells (SFC) per million PBMC, which increased to a geometric mean of 250 SFC (95% CI 107–583) at day 28 taking an average across all dose groups (Figure 3a). Responses were highest at day 28 in participants immunised with 2.5 × 10^10^ vp and were significantly increased after vaccination only in this dose group (*p* < 0.05, Kruskall-Wallis test with Dunn’s multiple comparison test compared with D0 responses).

Responses were detected to all antigens at day 28 with geometric mean responses ranging as follows; *AhpC*- 56 SFC, *Gsd*-41SFC, *p12*-64 SFC, *mpa*-52 SFC per million PBMC (Figure 3b).

### 3.4. Anti-Vector NAbs

**Figure 4:**
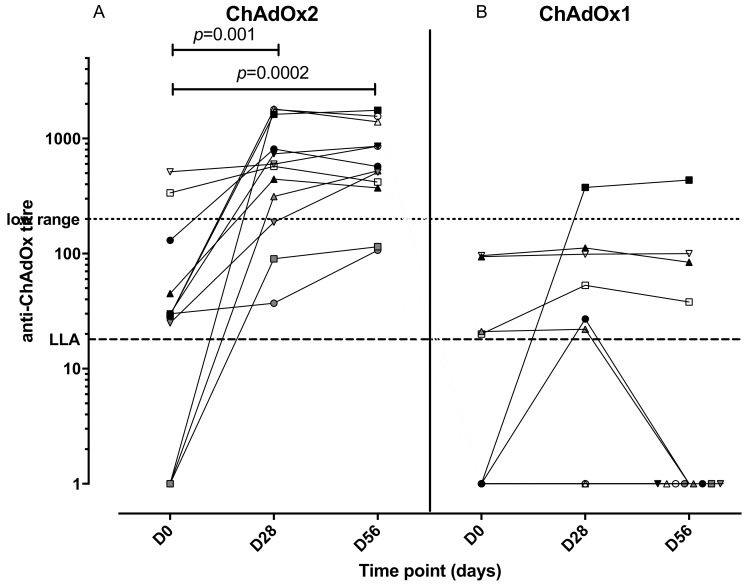
Anti-ChAdOx2 (**A**) and ChAdOx1 (**B**) vector neutralizing antibody titres at different timepoints (pre-enrolment, day 28 and day 56 post vaccination). LLA: lower limit of assay for accurate quantitation. Titres of neutralizing antibodies to ChAdOx2 increased significantly at day 28 and 56 compared with day 0 (Friedman test with Dunn’s multiple comparison test), but not to ChAdOx1.

**Figure 5:**
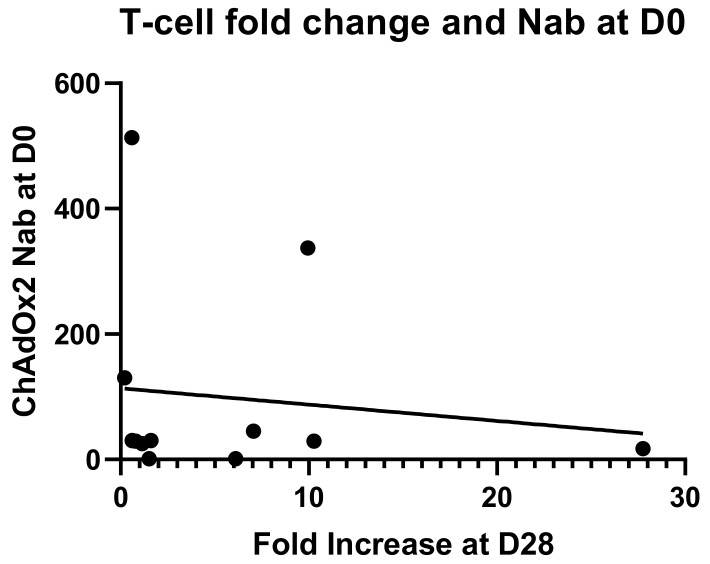
Linear regression for neutralising antibody titres against the vector at baseline and fold change in T-cell responses against the vaccine antigens at D28. No significant correlation has been found (*r^2^* = 0.01647, *p* = 0.6910).

An anti-vector neutralising antibody analysis was performed to establish whether volunteers enrolled in the study had pre-existing NAbs to ChAdOx2 or the related vector ChAdOx1 (Figure 4). Volunteers with neutralizing titres <1:18 in the SEAP assay were classified as negative, and titres of 1:18–200 were considered to be low, in accordance with other published clinical studies [35,42]. Two participants (of 12 tested) had higher levels of pre-existing NAbs to ChAdOx2 (titres 1:337 and 1:513) at D0 and both of them had had a ChAd63 vectored vaccine more than 12 months before enrolment. Seven participants had low level titres (3 of those had previously received a ChAd63 vectored vaccine) and 3 participants were seronegative (1 of those had previously received a ChAd3 vectored vaccine) for anti-ChAdOx2 NAbs prior to vaccination (titres <1:18). Following vaccination with ChAdOx2 HAV, all 3 seronegative participants seroconverted with NAb titres to ChAdOx2 ranging from 1:90 to >1:1800 at day 28. Titres to ChAdOx2 increased significantly after vaccination and remained significantly above baseline at D56 (Friedman test with Dunn’s multiple comparison test; *p* = 0.001 at day 28, *p* = 0.002 at day 56). The effect of ChAdOx2 dose on Nab titre was not assessed due to small group sizes. An association between pre-existing Nabs to ChAdOx2 at baseline and a failure to reach at least 4-fold increase in T-cell responses at D28 has not been confirmed, although the sample size is too small to reach any definitive conclusions (relative risk 2.1, 0.6915–11.71 95% confidence interval, Fisher’s exact test *p* = 0.5105, see Figure 5 for linear regression). Neutralising titres were also measured to ChAdOx1, another replication deficient adenoviral vector. Pre-existing titres to ChAdOx1 were only detected in 4 participants and were low. Responses did not increase significantly after vaccination at either D28 or D56 (Friedman test with Dunn’s multiple comparison test; *p* > 0.05).

## 4. Discussion and Conclusions

Adenovirus vectors are highly effective carriers for delivery of foreign antigens into host cells as they can elicit both specific antibodies and T cell responses [43]. Recombinant human and simian adenovirus vectored vaccines have now been extensively used in clinical trials of infectious diseases and cancer vaccines and thousands of volunteers have now been enrolled in studies of investigational vaccines against HIV, Malaria, Tuberculosis, Influenza and, most recently, Ebola [35,44,45,46,47].

T cell responses are known to be the main protective mechanism against intracellular pathogen’s infection and vaccination strategies against these organisms have traditionally focused on the use of whole live attenuated or inactivated microorganisms as a means of eliciting cellular immune responses. However, the use of replication competent live attenuated vaccines carries the risks of inadequate attenuation, potentially leading to disseminated disease particularly in immunocompromised hosts. The use of replication deficient vectors therefore avoids the risk of disseminated disease whilst maintaining the advantages of native antigen presentation, elicitation of T cell immunity and the ability to express multiple antigens [48].

This study was the first clinical use of the novel recombinant replication deficient chimpanzee adenovirus ChAdOx2 as a vaccine vector. The ChAdOx2 HAV vaccine was shown to be safe at the dose of 5 × 10^10^ vp, following a dose escalation approach using a ‘three-plus-three’ study design. The vaccine was well tolerated at the 3 doses tested with acceptable reactogenicity in the highest dose group. There were no serious adverse events reported and the vast majority of symptoms were mild in nature. All reported solicited and unsolicited AEs were self-limited and completely resolved in ≤4 days. The profile of adverse events reported in this trial is similar and comparable with other closely related simian adenovirus expressing several different antigens, such as ChAdOx1 and ChAd63 vectored vaccines [35,49]. This ChAdOx2 vectored vaccine was better tolerated and less reactogenic at the 5 × 10^10^ vp dose than its predecessor ChAdOx1 vector vaccine expressing influenza antigens where 3 out of 6 volunteers developed objective fevers and 2 of those also reported severe local and systemic AEs at the same dose [35].

T-cell responses were significantly higher than baseline at 1-month post vaccination with ChAdOx2 HAV in the 3 volunteers enrolled at the intermediate dose group (2.5 × 10^10^ vp) and 2 out of 6 volunteers in group 3 expressed 6–7 fold higher T-cell responses to the whole antigen at day 28 compared to baseline assessments at day 0. One explanation for the modest immunogenicity observed in the higher dose group could be the presence of anti-vector neutralising antibodies prior to vaccination, as 4 out of 6 volunteers enrolled in group 3 had previously received an adenovirus vectored vaccine encoding different antigens more than a year before enrolment. However, only 1 out of the 6 participants receiving the highest dose had higher titres against the ChAdOx2 vector at baseline (1:513) and 3 of the participants in the group had low titres at baseline ranging from 1:25 to 1:45. The development of recombinant simian adenovirus derived from rare serotypes was driven by the presence of high levels of pre-existing antibodies to human adenoviruses as vaccine vectors candidates [50]. Cross reactivity between human adenovirus vectors arising from natural infection is well established [51], however, the significance of prior humoral and cellular immunity against other simian adenovirus vectors, such as ChAdOx1 and ChAd63 for example, that could cross-react with ChAdOx2, has not been described. Here we show that there is considerable variability in the seroprevalence of responses to ChAdOx2 and ChAdOx1 before vaccination and that vaccination with ChAdOx2 induces significant increases in neutralizing antibody titre to the vector. However, despite widely reported cross-reactivity between human adenoviruses, induction of high titre neutralising antibodies to ChAdOx2 did not induce a detectable increase in titres to a related adenoviral vector ChAdOx1, suggesting that use of multiple viral vectors for different candidate vaccines will not be adversely affected by cross-reactive neutralising antibodies.

Prime-boost regimens have been shown to induce and maintain high levels of cellular immune responses [43], and therefore a second dose of ChAdOx2 HAV might be needed in order to achieve sustained T cell responses past 28 days post vaccination. Heterologous prime-boost vaccination schedules, where the same antigen is given by different viral vectors (e.g. Adenovirus prime with a Poxvirus boost), have previously shown to be more effective than homologous prime-boost strategies [52]. A new study assessing the safety and immunogenicity of a simian adenovirus prime (ChAdOx2) with a modified vaccinia Ankara vector boost (MVA) encoding the *AhpC*, *Gsd*, *p12* and *mpa* antigens could boost T-cell responses seen with the ChAdOx2 HAV prime.

In conclusion, the novel replication deficient chimpanzee adenovirus vector ChAdOx2 encoding antigens from the *Mycobacterium avium paratuberculosis* is safe, well tolerated and modestly immunogenic in healthy adult volunteers.

## Supplementary Materials

The following are available online at https://www.mdpi.com/2076-393X/7/2/40/s1, Supplementary Figures S1–S6: Proportion of volunteers reporting local and systemic AEs with ChAdOx2 HAV at different doses; Supplementary Table S1: Unsolicited AEs considered possibly, probably or definitively related with ChAdOx2 HAV; Supplementary Table S2: Transient laboratory AEs.
