# Supplementary material for: Safety and Immunogenicity of a Novel Recombinant Simian Adenovirus ChAdOx2 as a Vectored Vaccine
Source: Vaccines (Basel). 2019 May 15;7(2):40. doi: 10.3390/vaccines7020040 (PMC6630572; doi:10.3390/vaccines7020040)
Supplement: Supplementary file 1 [file vaccines-07-00040-s001.pdf]

Supplementary Material – Figures and Tables

Adverse Events – ChAdOx2 HAV

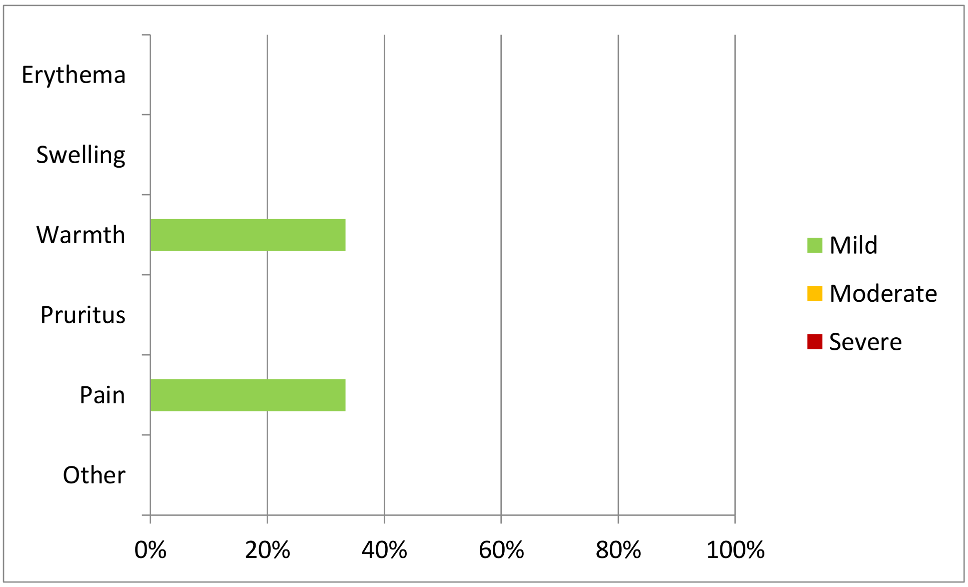

Suppl. Fig. 1: Proportion of volunteers reporting local AEs with ChAdOx2 HAV at the 5 x 10^9 vp dose

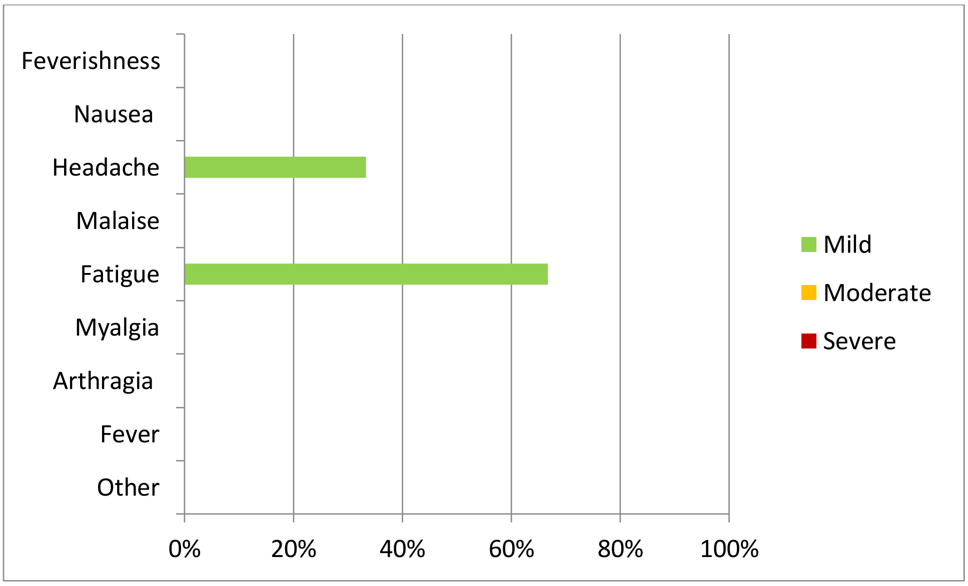

Suppl. Fig. 2: Proportion of volunteers reporting systemic AEs with ChAdOx2 HAV at the 5 x 10^9 vp dose

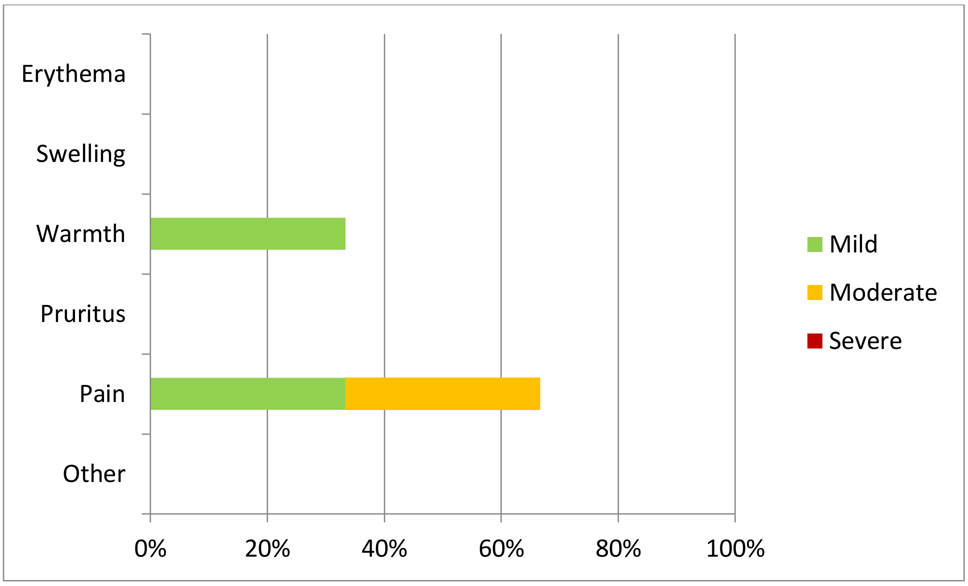

Suppl. Fig. 3: Proportion of volunteers reporting local AEs with ChAdOx2 HAV at the 2.5 x 10^{10} vp dose

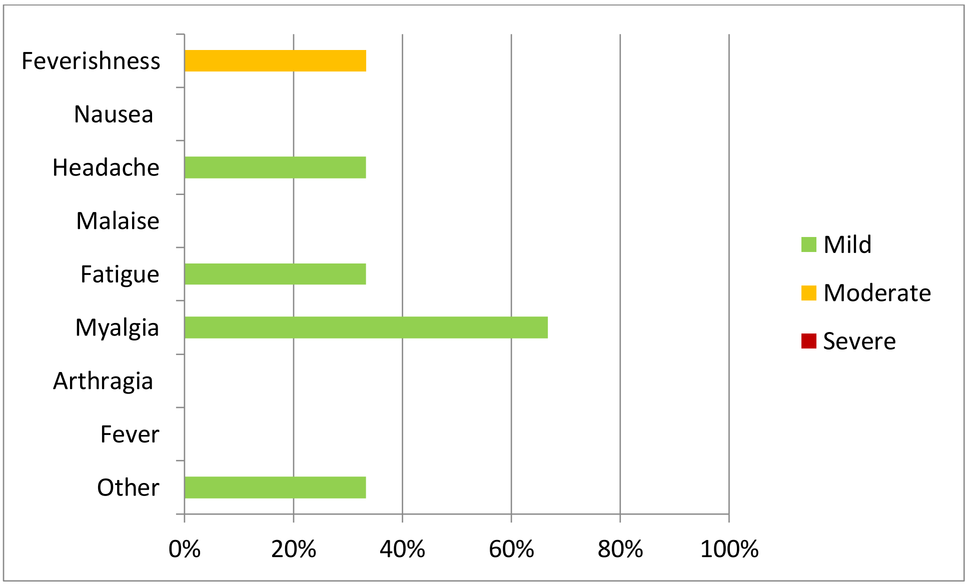

Suppl. Fig. 4: Proportion of volunteers reporting systemic AEs with ChAdOx2 HAV at the 2.5 x 10^{10} vp dose

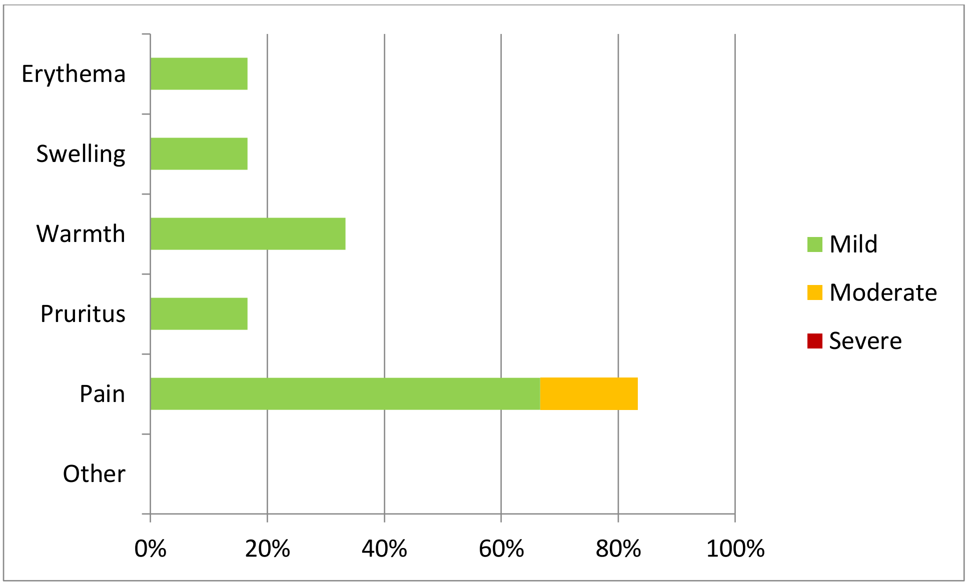

Suppl. Fig. 5: Proportion of volunteers reporting local AEs with ChAdOx2 HAV at the 5 x 10^{10} vp dose

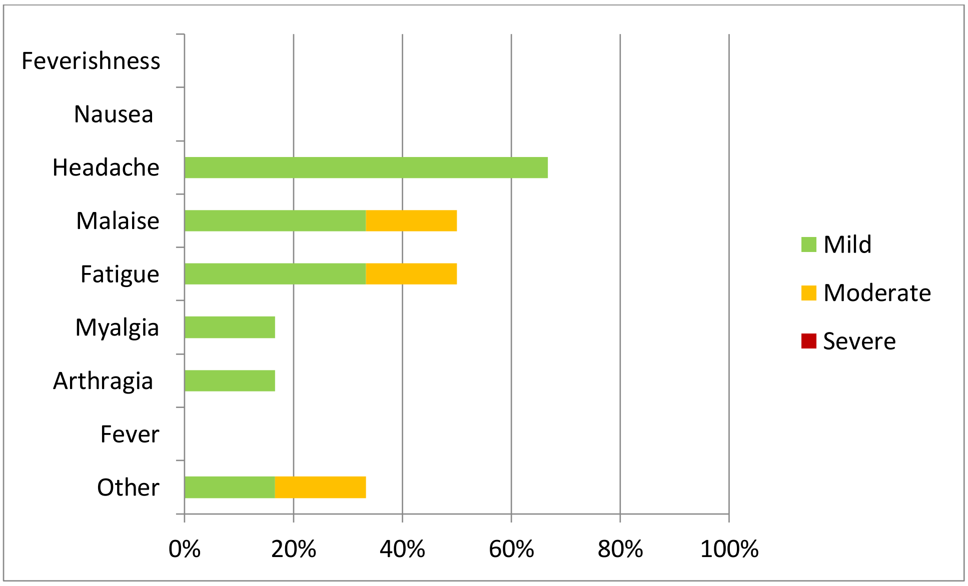

Suppl. Fig. 6: Proportion of volunteers reporting systemic AEs with ChAdOx2 HAV at the 5 x 10^{10} vp dose.

Suppl. Table 1. Unsolicited AEs considered possibly, probably or definitively related with ChAdOx2 HAV in HAV001

Id	Group	Days since vaccination	Duration of AE (days)	Description (Meddra PT)	Associated medication	Maximum severity
00101008	2.5×10^{10}	0	2	Paraesthesia	No	Mild
00101008	2.5×10^{10}	10	1	Vaccination site pain	Yes	Moderate
00101011	5×10^{10}	7	2	Malaise	Yes	Moderate
00101011	5×10^{10}	7	2	Oropharyngeal pain	Yes	Moderate
00101011	5×10^{10}	7	2	Fatigue	Yes	Moderate
00101021	5×10^{10}	6	3	Throat irritation	No	Mild
00101021	5×10^{10}	6	2	Sneezing	No	Mild
00101021	5×10^{10}	7	1	Eye pruritus	No	Mild

Suppl. Table 2. Transient laboratory AEs in HAV001.

Id	Group	Event	Severity	Timepoint
00101004	2.5×10^{10}	Lymphopaenia	Mild	D2
00101011	2.5×10^{10}	Lymphopaenia	Mild	D2
00101018	5×10^{10}	Neutropaenia	Mild	D2
00101020	5×10^{10}	Thrombocytopaenia	Moderate	D2
